# Genome Sequence Comparisons between Small and Large Colony Phenotypes of Equine Clinical Isolates of *Arcanobacterium hippocoleae*

**DOI:** 10.3390/ani14111609

**Published:** 2024-05-29

**Authors:** Lisanework E. Ayalew, Zelalem H. Mekuria, Beatrice Despres, Matthew E. Saab, Shivani Ojha

**Affiliations:** 1 Atlantic Veterinary College, University of Prince Edward Island (UPEI), 550 University Ave, Charlottetown, PE C1A 4P3, Canada; 2 Global One Health Initiative (GOHI), The Ohio State University (OSU), Columbus, OH 43210, USA; mekuria.3@osu.edu; 3 Department of Veterinary Preventative Medicine, College of Veterinary Medicine, The Ohio State University (OSU), Columbus, OH 43210, USA

**Keywords:** *A. hippocoleae*, NGS, Oxford Nanopore, mares, reproductive tract

## Abstract

**Simple Summary:**

Understanding the genetic makeup of microorganisms provides information on their evolutionary relationship with other similar organisms. It also sheds light on their ability to cause disease in susceptible individuals and to resist the hosts’ defense mechanisms, including the effects of antimicrobial therapy. In this study, we performed complete genome sequencing and characterization of bacteria called *Arcanobacterium hippocoleae,* which were isolated from the reproductive tract of infected mares. These bacteria displayed small and large phenotypes when grown on artificial media. The whole genome sequences between the large phenotypes were closely related, while the small and large types were distant from each other in terms of sequence size and identity. Putative genetic elements that might be associated with disease conditions were identified in both bacterial phenotypes. Several genes that express hypothetical proteins with unknown functions were also detected. This study provides the complete genetic structure and analysis of different phenotypes of *Arcanobacterium hippocoleae* and will serve as a benchmark for future studies to identify the potential function and role of the bacterial proteins in the ability of the bacterium to produce reproductive tract diseases in mares.

**Abstract:**

*Arcanobacterium hippocoleae* is a Gram-positive fastidious bacterium and is occasionally isolated from the reproductive tract of apparently healthy mares (*Equus caballus*) or from mares with reproductive tract abnormalities. Apart from a few 16S rRNA gene-based GenBank sequences and one recent report on complete genome assembly, detailed genomic sequence and clinical experimental data are not available on the bacterium. Recently, we observed an unusual increase in the detection of the organism from samples associated with mare reproductive failures in Atlantic Canada. Two colony morphotypes (i.e., small, and large) were detected in culture media, which were identified as *A. hippocoleae* by MALDI-TOF mass spectrometry and 16S rRNA gene sequencing. Here, we report the whole genome sequencing and characterization of the morphotype variants. The genome length of the large phenotypes was between 2.42 and 2.43, and the small phenotype was 1.99 Mbs. The orthologous nucleotide identity between the large colony phenotypes was ~99%, and the large and small colony phenotypes was between 77.86 and 78.52%, which may warrant the classification of the two morphotypes into different species. Phylogenetic analysis based on 16S rRNA genes or concatenated housekeeping genes grouped the small and large colony variants into two different genotypic clusters. The UvrA protein, which is part of the nucleotide excision repair (NER) system, and 3-isopropoylmalate dehydratase small subunit protein expressed by the *leuD* gene were identified as potential virulence factors in the large and small colony morphotypes, respectively. However, detailed functional studies will be required to determine the exact roles of these and other identified hypothetical proteins in the cellular metabolism and potential pathogenicity of *A. hippocoleae* in mares.

## 1. Introduction

*Arcanobacterium hippocoleae* was first isolated from a vaginal discharge sample from a mare (*Equus caballus*) and was assigned to the genus *Arcanobacterium* based on phenotypic and genetic characterization of the organism [1]. Phenotypically, it is a non-spore-forming, non-acid fast, facultatively anaerobic, Gram-positive, irregularly shaped, non-branching rod-shaped bacterium [1]. So far, there are very few reports on the isolation and identification of the bacterium. The isolates were from the reproductive tract of apparently healthy mares or from mares that developed placentitis and stillbirth. The organism appears to be adapted to the reproductive tract of horses, but its pathogenic significance is unclear. The second report on *A. hippocoleae* was made in 2003. The bacterium was isolated in pure culture from a urine sample from a horse and identified by 16S rRNA gene sequencing [2]. The health status of the animal was not described in the report. The third report on *A. hippocoleae* was associated with placental lesions in a mare, and large numbers of bacteria were isolated from the placenta, as well as in the lung and the stomach contents of a late-term stillbirth American Quarter Horse foal [3]. The fourth report was based on the culture and isolation of the bacterium from a swab sample collected from an apparently healthy mare [4]. In 2019, isolation and characterization of *A. hippocoleae* were reported from the genital swabs of 15 apparently healthy mares [5]. Out of the eighteen isolates reported, sixteen isolates have been characterized by a combination of Matrix-Assisted Laser Desorption/Ionization–Time of Flight mass spectroscopy (MALDI-TOF MS), 16S rRNA gene sequencing, and biochemical tests [4,5]. In the latest case report made in Brazil in 2022, *A. hippocoleae* was isolated from the uterus of a mare with unexplained reproductive failure and an oscillating reproductive history [6].

Understanding the role of *A. hippocoleae* as a commensal in the microbiome of the mare’s genital tract or its significance as a pathogen is very important. Currently, there are only a few metagenomic-based reports available focusing on the reproductive system of mares [7,8,9,10,11]. However, all these studies were based on 16S rRNA gene sequencing, and their resolutions were only up to the genus level.

In the last two years, there has been an unusually increased frequency of isolation and identification of *A. hippocoleae* from the reproductive tract of mares in Atlantic Canada associated with a history of reproductive problems. In most cases, *A. hippocoleae* were isolated alone or in mixed growth with other bacteria. Currently, sequence-based information or experimental clinical data are not available regarding the pathogenicity of *A. hippocoleae.* Therefore, the objectives of this study were to (1) provide whole genome-based sequence comparisons between two morphotypes of *Arcanobacterium* species, which were isolated from mares with reproductive tract abnormalities and identified as *A. hippocoleae* by MALDI-TOF MS, and (2) to provide sequence-based information regarding virulence factors, antimicrobial resistance genes, and the evolutionary relationship of the bacterial isolates with other *Arcanobacterium* species.

## 2. Materials and Methods

### 2.1. Bacteria Isolation and Identification

Uterine lavage and swab samples were aseptically collected from Standardbred and Hanoverian mares with reproductive problems (Supplementary Table S1) and transported to the Atlantic Veterinary College (AVC) Diagnostic Services Bacteriology Laboratory (DSBL) for bacterial isolation and identification. Five samples collected from five different mares were submitted between March and May of 2022. The samples were inoculated onto Columbia agar with 5% sheep blood and incubated at 35 °C with 5% CO_2_ for 48 h. Bacterial growth was identified by MALDI-TOF MS using the Bruker microflex LT and MBT Compass reference library v11 (Bruker Daltonic, Billerica, MA, USA).

### 2.2. Bacterial Genomic DNA Extraction and Analysis

A single bacterial colony was transferred from each blood agar plate to 5 mL of brain heart infusion (BHI) broth with 10% fetal calf serum and grown at 35 °C for 48 h. A total of 1.5 mL of bacterial broth culture was centrifuged at 3000× *g* rpm for 10 min. The supernatant was discarded, and the pellet was subjected to DNA extraction using the GenElute^TM^ Bacterial Genomic DNA kit (Sigma-Aldrich, St. Louis, MO, USA) as per the company’s protocol. The purity and concentration of the purified DNA were evaluated by a Nanophotometer (Implen, Munich, Germany) and Qubit fluorometer (Invitrogen, Waltham, MA, USA), respectively. The molecular size of the purified genomic DNA was analyzed by agarose gel electrophoresis.

### 2.3. MinION Sequence Library Preparation and Sequencing

The sequence library was prepared using the Ligation Sequencing gDNA (SQK-LSK110) kit (Oxford Nanopore Technologies Ltd., Oxford, UK) following the company’s protocol. Briefly, the bacterial genomic DNA (1 µg total) was end repaired using the NEBNext FFPE DNA repair mix and NEBNext Ultra II end repair/dA tailing module (New England Biolabs Ltd., Ipswich, MA, USA). The end-repaired DNA was cleaned up using AMPure XP beads (Beckman Coulter Life Sciences, Brea, CA, USA). Adaptor ligation was performed using the NEBNext quick ligation module (New England Biolabs Ltd.) using the Short Fragment Buffer (SFB) (Oxford Nanopores) followed by DNA clean up as described before. The DNA libraries were loaded onto primed R9.4.1 flow cells (FLO-MIN106) and sequenced in a MinION Mk1B sequencer (Oxford Nanopore Technologies Ltd.) connected to the MinKNOW software version 24.02.6. The raw reads generated by MinKNOW were base called using Guppy version 6.4.8 (Oxford Nanopore Technologies Ltd.) with the “Fast” configuration of the algorithm with a default quality filter of 7. Total yield, mean quality, and mean read length of passed reads were determined using MinIONQC version 1.4.2 (Oxford Nanopore Technologies Ltd.).

### 2.4. DNA Sequence Assembly and Analysis

Passed Fastq files were imported into Geneious Prime software 2024.0.4 (Dotmatics, Boston, MA, USA), and the trimmed raw reads were assembled de novo using the Flye assembler plugin [12,13] with the minimum overlap length and the minimum contig assembly coverage set at 3000 and 100, respectively. Assembly was performed through 3 polishing iterations. The quality of the final assemblies was examined by the Quast [14] and CheckM [15,16] programs. SpeciesFinder (Center for Genomic Epidemiology, Technical University of Denmark, 2800 Kongens Lyngby, Denmark) was used to predict the identity of the bacterial species. The similarity between the three genome sequences, including *A. hippocoleae* strain DSM 15539 (GenBank Reference# NZ_JAVDUJ010000001), were analyzed by the Orthologous Average Nucleotide Identity (OrthoANI) and original ANI Tools using OAT v0.93.1 software [17]. Further comparison of the genome sequences was performed by the Dot-Plot method using re-DOT-able software version 1.2 (Babraham Bioinformatics, Cambridge, UK) with a window size of 19. In addition, genome alignment was performed in Geneious Prime software (Dotmatics) using the progressiveMauve plugin and Large-Scale Genome Alignment Tool (LASTZ) [18] with default parameters. The genomes were submitted to the all-bacterial bioinformatics database and analysis resource center [19], and coding sequence (CDS) and other motifs were annotated by the Rapid Annotations using the Subsystems Technology tool kit (RASTtk) server [20,21,22,23] with genetic code 11. Comparison of the annotated protein sequences of the three bacterial genomes was performed using the *A. hippocoleae* strain DSM 15539 as a reference by the BV-BRC’s proteome comparison tool [21] with default parameters. Similar protein sequence comparisons were performed using the sequences of our isolates and different *Arcanobacterium* species. Subsystem pathway predictions were performed using the Pathosystems Resource Integration Center (PATRIC) database [24]. A circa plot was used to show the distribution of the genome annotations in each genome. Homology to antimicrobial resistance genes (AMRs) was examined by the k-mer-based AMR gene detection method in EPI2ME (Oxford Nanopore Technologies Ltd.) and PATRIC [19], employing the Comprehensive Antibiotic Resistance Database (CARD), National Database of Antibiotic Resistant Organisms (NDARO), and DrugBank. Homology to known virulence genes was analyzed using the PATRIC-VF database [25] and the virulence factor database (VFDB) [26].

### 2.5. Phylogenetic Analysis

The phylogenetic relationship between the three genomes, including ten other bacterial reference genomes selected from the National Center for Biotechnology (NCBI) database in PATRIC, was analyzed using the codon tree service in BV-BRC. Briefly, the amino acid and nucleotide sequences from twenty BV-BRC global Protein Families (PGFams) [24] with the highest alignment score were selected. The codon tree service aligns concatenated protein-coding sequences using the Codon_align function in BioPython [27] and constructs a phylogenetic tree using RaxML [28]. The phylogenetic tree was viewed in iTOL V5 [29]. Additionally, eighty-two complete genome sequences of different *Arcanobacterium* species available in GenBank were used to construct a phylogenetic tree, with the sequences of our isolates using the codon tree method as described above. In addition, phylogenetic analysis was performed in Geneious Prime software (Dotmatics) based on the 16S rRNA gene of our isolates plus 16S rRNA gene sequences of *A. hippocoleae* retrieved from GenBank. The genes were aligned using MAFFT [30], and the phylogenetic tree was constructed using RAxML [28].

## 3. Results

### 3.1. Bacteriology

*A. hippocoleae* were isolated from the reproductive tract of five mares, including two Standardbred, one Thoroughbred, and one Hanoverian mare, and the breed of the last mare was not specified during sample submission (Supplementary Table S1). Four of the mares were from Prince Edward Island (PEI), while the Thoroughbred mare was from Nova Scotia, Canada. One of the Standardbred mares (Stan) was inseminated with fresh semen and was 283 days pregnant. The fetus was hyperactive, and the mare had vaginal discharge and udder edema. The Hanoverian mare (Han) had a history of placentitis and premature foaling in the previous year and was treated with local and systemic antibiotics. When the mare began cycling a year later, it had purulent discharge, and a small amount of hyperechoic material was detected in the uterus upon ultrasound examination. No clinical history was available for the second Standardbred mare. The last sample was a pre-breeding swab from a mare that was bred by artificial insemination (AI) in the previous year but did not conceive. After 48 h of incubation, slow-growing, smooth, and small or large gray-colored colonies were observed. The colonies were non-hemolytic. MALDI-TOF MS analysis identified both colony morphotypes as *A. hippocoleae* with a high-confidence score value of ≥2. *A. hippocoleae* appeared as the primary organism, showing light to moderate growth in samples from four of the mares. Scant growth (single or two colonies) of *Streptococcus equi* subsp. *zooepidemics* was observed in three Standardbred mares, while moderate growth of *S. equi* subsp. *zooepidemicus* and *A. hippocoleae* was observed from the vulval discharge sample of the Hanoverian mare. *A. hippocoleae* was the sole organism isolated as moderate growth from the vaginal discharge of a Thoroughbred mare from Nova Scotia, Canada. The two large colony phenotypes isolated from one of the Standardbred mares and the Hanoverian mare were designated as Stan-Large and Han-Large, respectively. The small colony variant isolated from the Standardbred mare was designated as Stan-Small. The three isolates were subjected to whole genome sequencing.

### 3.2. Run Summary and Genome Assembly Statistics

The average total base-called data generated after nanopore sequencing was 2.76 Gb. The average quality score and average sequence length of raw reads were 11.2 and 4.3 Kb, respectively. Based on the EPI2ME Fastq WIMP (Oxford Nanopore Technologies Ltd.) analysis, all three bacterial isolates were classified under the family *Actinomycetaceae* and in the genus *Arcanobacterium*. A summary of the assembly details and genome quality is provided in Supplementary Table S2.

### 3.3. Genome Annotation and Subsystem Analysis

**Figure 1:**
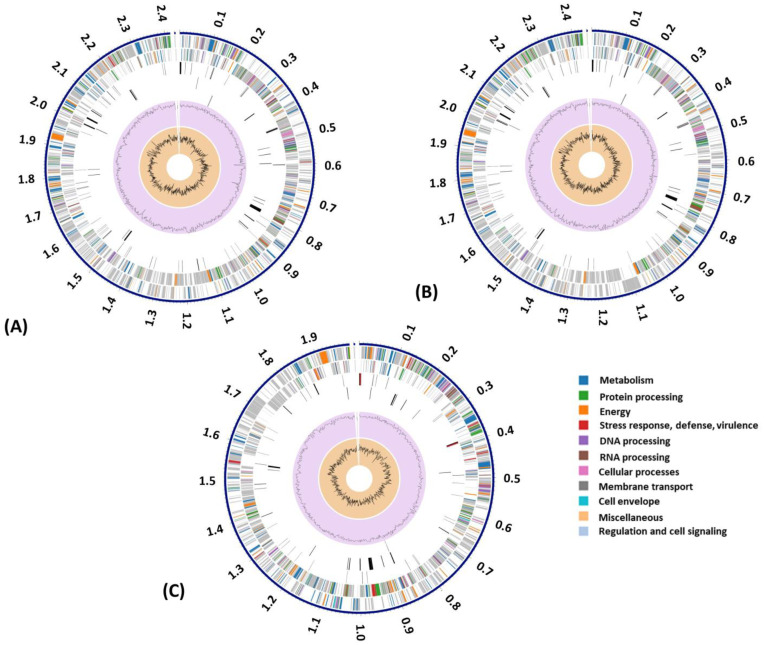
A circular graphical display of the distribution of the genome annotations of the Han-Large (**A**), Stan-Large (**B**), and Stan-Small (**C**) isolates of *A. hippocoleae*. This includes, from outer to inner rings, the contigs, CDS on the forward strand, CDS on the reverse strand, RNA genes, CDS with homology to known antimicrobial resistance genes, CDS with homology to known virulence factors, GC content, and GC skew. The colors of the CDS on the forward and reverse strands indicate the subsystem that these genes belong to.

Each genomic DNA was annotated using RASTtk in PATRIC. The number of identified protein-coding sequences, repeat regions, transfer RNA (tRNA), and ribosomal RNA (rRNA) genes for the genome of each bacterial isolate is summarized in Table 1. A circa plot displaying annotations of the Han-Large (Figure 1A), Stan-Large (Figure 1B), and Stan-Small (Figure 1C) genomes is shown. The number of proteins with functional assignments was 1435, 1327, and 1273 for the Han-Large, Stan-Large, and Stan-Small isolates, respectively.

**Figure 2:**
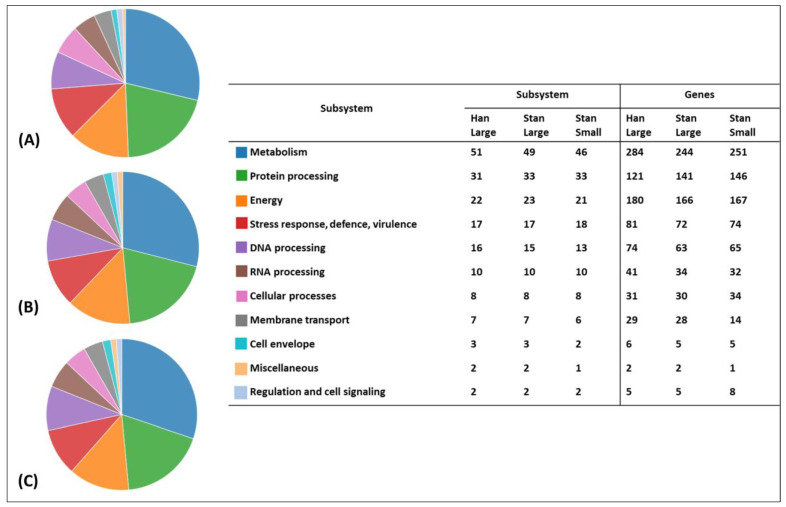
An overview of the subsystems for the genomes of the Han-Large (**A**), Stan-Large (**B**), and Stan-Small (**C**) isolates of *A. hippocoleae*.

The number of Enzyme Commission (EC) [31] and Gene Ontology (GO) [32] assignments for each bacterial genome, including the number of proteins with pathway assignments based on mapping to the Kyoto Encyclopedia of Genes and Genomes (KEGG) pathways database [33], are described in Table 1. More hypothetical proteins were identified in the genomes of the Han-Large and Stan-Large isolates than in the genome of the Stan-Small isolate. An overview of the analysis of the subsystems for the genome of each bacterial isolate is shown in Figure 2A–C. Most of the proteins are identified to be involved in cellular metabolism followed by protein processing, energy generation, stress response, defense, virulence, DNA processing, RNA processing, and other miscellaneous proteins (Figure 2). The alignment of the second contig of the three isolates showed 100% nucleotide identity. The NCBI Nucleotide BLAST of the contig revealed a sequence identity of 99.83% with pECQ4552_IHU08 plasmid DNA identified in the *Escherichia coli* strain Q4552 (GenBank accession# CP077071.1). The identified plasmid DNA contains genes that express phage holin/antiholin component S and phage endopeptidase Rz proteins, among other hypothetical proteins.

### 3.4. Dot-Plot Analysis and Whole Genome Alignment

**Figure 3:**
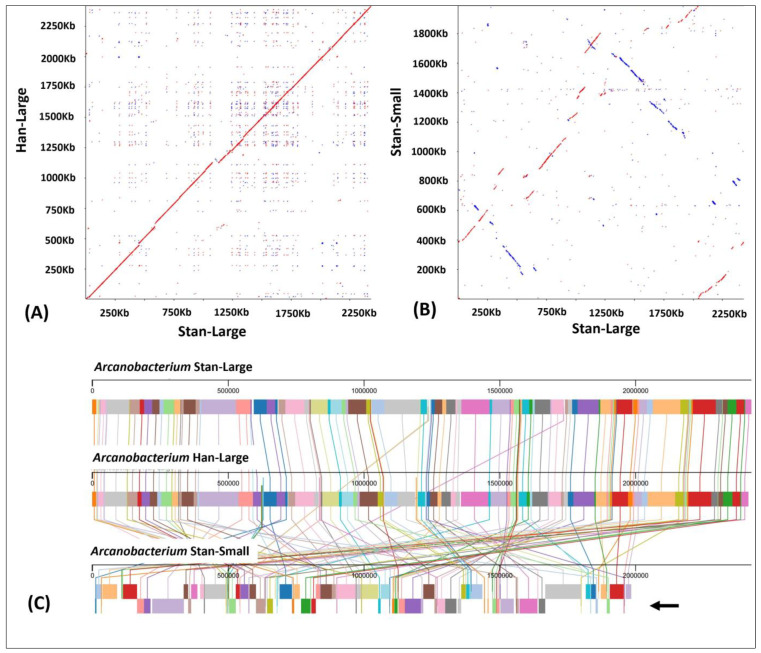
Dot-Plot analysis of the genomes of the Han-Large and Stan-Large isolates (**A**) and the Stan-Large and Stan-Small (**B**) isolates of *A. hippocoleae*. (**C**) Comparison of the genomic organization of the genomes of the Han-Large, Stan-Large, and Stan-Small isolates of *A. hippocoleae* by progressiveMauve alignment. Linear collinear blocks (LCB)s are shown by different colors. LCBs indicated by the arrow have an inverted orientation in the genome of the Stan-Small isolate.

A continuous match was observed when the genomes of the Stan-Large and Han-Large genomes were compared by a Dot-Plot analysis (Figure 3A). In contrast, deletions and inversions were observed when the genomes of the large phenotypes were compared with the Stan-Small genome (Figure 3B). Furthermore, progressiveMauve-based genome alignment was performed to determine the collinearity of the Stan-Large, Han-Large, and Stan-Small genomes. As indicated in Figure 3C, 101 linear collinear blocks (LCBs) were identified with genome rearrangements, inversions, and gene losses observed in the Stan-Small genome.

**Figure 4:**
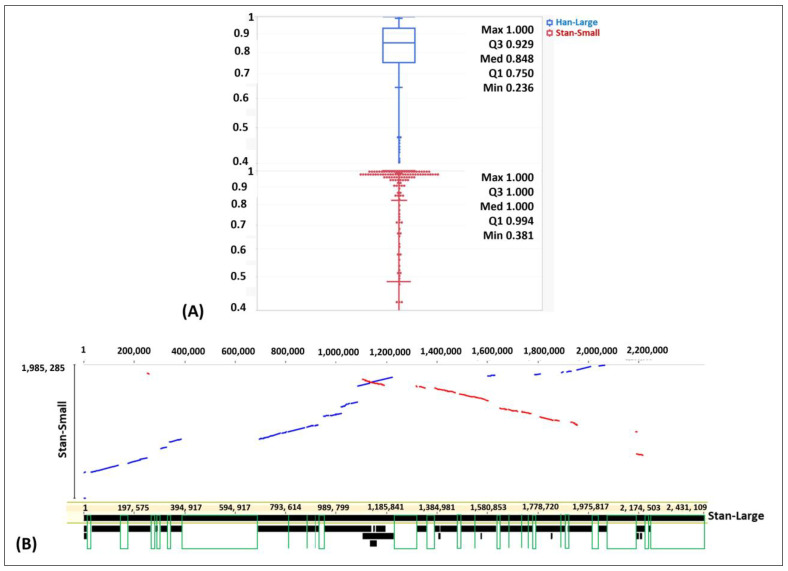
(**A**) Box plot showing the distribution of the percent nucleotide identity of genes with functional annotation between the genomes of the Stan-Large and Han-Large (shown in blue) as well as the Stan-Large and Stan-Small (shown in red) isolates of *A. hippocoleae*. (**B**) Large-Scale Genome Alignment (LASTZ) graph demonstrating the pairwise alignment of the genomes of the Stan-Large and Stan-Small *A. hippocoleae* isolates. Blue and red lines: forward and reverse gene orientations in the Stan-Small genome in comparison to the Stan-Large genome, respectively. Green boxes: areas of deletion in the Stan-Small genomes in comparison to the Stan-Large genome.

A box plot was generated using JMP V.17.1 to determine the distribution of the percent nucleotide identity of genes with functional annotations of the genomes of the Han-Large and Stan-Small *A. hippocoleae* isolates in comparison to the Stan-Large *A. hippocoleae* isolate. In the Han-Large genome, 50% of the coding sequence (CDS) had 100% sequence identity, and over 75% of the genes had more than 99% sequence identity with the corresponding CDSs of the Stan-Large genome (Figure 4A). On the contrary, in the Stan-Small genome, 50% of the CDSs had more than or equal to 85% sequence identity, and 75% of the CDSs had greater than or equal to 75% sequence identity in comparison to the corresponding CDS of the Stan-Large genome (Figure 4A). A pairwise Large-Scale Genome Alignment of the whole genomes of the Stan-Large and Stan-Small *A. hippocoleae* isolates demonstrated several areas of gene deletions in the Stan-Small genome compared to the Stan-Large genome (Figure 4B).

### 3.5. Antimicrobial Resistance (AMR) Genes and Potential Drug Targets

Several potential antibiotic resistance genes in the genome sequences of the three isolates were identified, including the AMR mechanisms, using different databases (Supplementary Table S3). Most of the genes detected were associated with rRNA mutations, and the rest were identified through the protein homolog and protein variant models. However, the EPI2ME Fastq antimicrobial resistance gene analysis indicated that none of the identified genes were clinically relevant.

### 3.6. Sequence Comparison and Phylogenetic Tree Analysis

**Figure 5:**
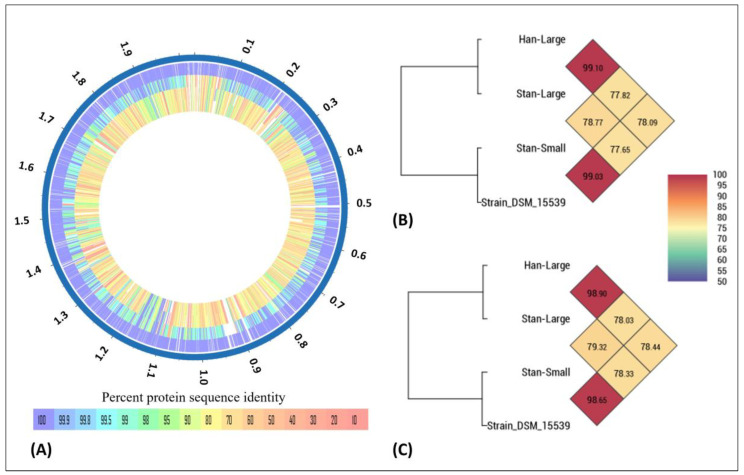
(**A**) Percent protein sequence identity between the genomes of the Han-Large, Stan-Large, and Stan-Small isolates of *A. hippocoleae* using the sequence of *A. hippocoleae* strain DSM 15539 as a reference. List of tracks from outside to inside: *A. hippocoleae* strain DSM 15539, Stan-Large, Han-Large, and Stan-Small. (**B**) OrthoANI and (**C**) ANI values between *A. hippocoleae* strain DSM 15539 and the Han-Large, Stan-Large, and Stan-Small isolates of *A. hippocoleae*. The tree scale represents the percent average nucleotide identity.

A comparison of the coding sequences of the hypothetical proteins and proteins with functional assignments of the three isolates indicated that the orthologous average nucleotide identity between Han-Large and Stan-Large was 99.1%, and the average nucleotide identity between Stan-Large and Stan-Small, and Han-Large and Stan-Small was 78.77% and 77.82%, respectively (Figure 5A,B). In addition, protein sequence alignment and genome of sequences of our isolates were compared with the genome of *A. hippocoleae* strain DSM 15539 retrieved from GenBank. The orthoANI between Stan-Small and strain DSM 15539 was 99.03%. The orthoANI between Stan-Large and Han-Large, and strain DSM 15539 was 78.33 and 78.44%, respectively (Figure 5B). Original ANI values are shown in Figure 5C. Moreover, a protein sequence comparison of our isolates, *A. hippocoleae* strain DSM 15539 and six other *Arcanobacterium* species from GenBank, indicates that the Stan-Small sequence is closely related to strain DSM 15539 followed by the Stan-Large and Han-Lage sequences compared to the protein sequences of the other *Arcanobacterium* species (Supplementary Figure S1).

**Figure 6:**
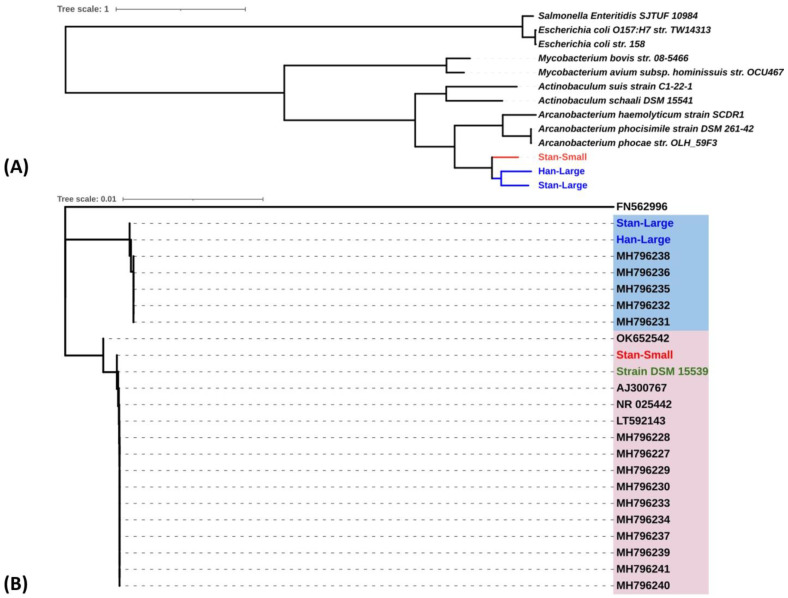
(**A**) Phylogenetic tree analysis based on the concatenated sequences of twenty protein-coding regions with the highest alignment score and (**B**) the16S rRNA gene sequences. The large and the small colony variants of *A. hippocoleae* isolates are shown in blue and red fonts, respectively. The *A. hippocoleae* strain DSM 15539 is shown in a green font. The remainder of the light blue and pink highlighted groups show separate clusters of different *A. hippocoleae* isolates based on 16S rRNA gene sequences. The isolates are indicated by the GenBank sequence accession numbers. *Arcanobacterium phocisimile* (FN562996) was used as an outgroup.

**Figure 7:**
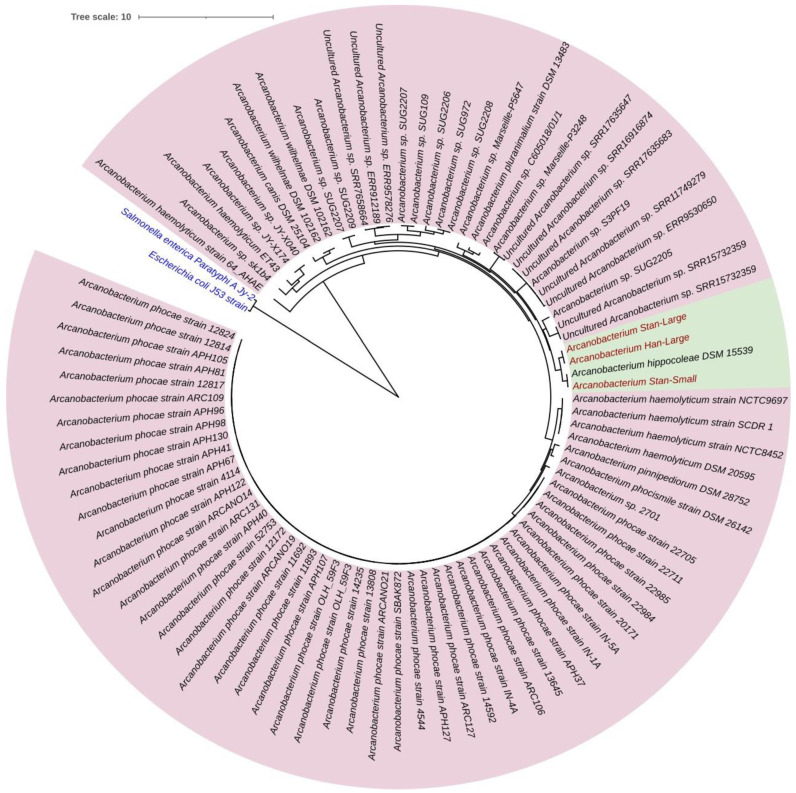
Phylogenetic tree constructed by the BV-BRC codon tree pipeline using the whole genomes of eighty-two isolates of different *Arcanobacterium* species, including the genomes of our three isolates. The clustering of our isolates and *A. hippocoleae* strain DSM 15539 are highlighted in light green. Our isolates are shown in red fonts. *Salmonella enterica* serovar Paratyphi A Jy-2 strain and *Escherichia coli* J53 strain shown in blue fonts were used as outgroups.

Phylogenetic tree analysis based on the concatenated sequences of twenty protein-coding regions (Table 2) with the highest alignment score of our three isolates, including ten other bacterial species retrieved from GenBank, indicated that the Stan-Large and Han-Large isolates clustered together, while the Stan-small isolate clustered in a different group (Figure 6A). Similarly, phylogenetic analysis based on the 16S rRNA gene sequences of our isolates together with the 16S rRNA gene sequences of twenty *A. hippocoleae* retrieved from GenBank showed that the *A. hippocoleae* sequences clustered into two different genotypic groups, and the Stan-Large and Han-Large isolates clustered in the same group with a 99.94% sequence identity, while Stan-Small and *A. hippocoleae* strain DSM 15539 clustered together in a separate group with a 100% sequence identity (Figure 6B). The 16S rRNA gene sequence identity between Stan-Large and Han-Large with *A. hippocoleae* strain DSM 15539 were 99.02 and 98.95%, respectively. In addition, the whole genome-based phylogenetic tree construction of eighty-two isolates of different Arcanobacterium species together with the sequences of our isolates revealed that Stan-Large and Stan-Small subclustered together, while Stan-Small and *A. hippocoleae* strain DSM 15539 grouped together in a separate subcluster. However, all three isolates were evolutionarily closely related to *A. hippocoleae* strain DSM 15539 compared to the other *Arcanobacterium* species (Figure 7).

## 4. Discussion

Despite the isolation of *A. hippocoleae* from the reproductive tract of mares with some related health issues [1,2,3,4,5,6], the significance of the bacterium as a commensal, primary, or opportunistic pathogen has not been established. The genus *Arcanobacterium* was detected in 16S rRNA gene sequence-based metagenomic studies with low relative abundance from the uterus and vagina of healthy mares [7,9,11]. However, metagenomic techniques that provide species-level information are required to confirm whether *A. hippocoleae* is part of the normal microbial ecology of the genital tract of mares. In this study, we provided a comprehensive whole genome comparison of two morphotypes of *A. hippocoleae* clinical isolates for the first time. Although clinically relevant antimicrobial resistance genes were not identified, potential virulence genes, which may aid bacterial survival and proliferation in the mare’s reproductive tract, have been identified. In addition, we established the evolutionary relationship between the two morphotypes of *A. hippocoleae* and between our *A. hippocoleae* isolates and other *Arcanobacterium* species at the whole genome level.

Previously, the diagnostic bacteriology laboratory rarely identified *Arcanobacterium* species from horses. Any such identified organisms were either very low in number to be clinically significant or were accompanied by a predominant reproductive tract pathogen, such as *S. zooepidemicus*. However, the five sequential cases in mares clustered in a period of a few months in the summer of 2022 were noticeable since all the mares showed clinical signs of reproductive tract disease and *A. hippocoleae* was detected in tandem with the absence of other known reproductive tract pathogens. These observations prompted us to sequence and characterize the whole genome of the small and large colony morphotypes (i.e., isolated from the Standardbred [Stan-Small and Stan-Large] and Hanoverian [Han-Large] breeds of mares) of *A. hippocoleae*.

Both the small and large colony phenotypes were identified as *A. hippocoleae* in an NCBI blast of the respective 16S rRNA genes, which was consistent with the type of identification by MALDI-TOF mass spectrometry. Nevertheless, phylogenetic analysis based on the 16S rRNA gene or 20 concatenated housekeeping genes clustered the large and small phenotypes into two distinct sublineages, indicating intraspecies genotypic differences between the small and large colony variants of the bacteria. Previous studies also reported the existence of two sublineages of *A. hippocoleae* based on 16S rRNA gene sequencing of 15 bacterial isolates [5]. However, the study did not provide information on the association between colony morphology and genotypic characteristics. The phenotypic description of *A. hippocoleae* isolated from the uterus of a Thoroughbred mare with reproductive failure [6] was similar to the phenotypic characteristics of Stan-Small, and the two were clustered in the same genotypic group based on the 16S rRNA gene sequence.

The genome of the small colony phenotype of *A. hippocoleae* was relatively smaller than the genomes of the large colony phenotypes with demonstrable inversions and deletions. This might be associated with a gain or loss of non-essential genetic elements for survival and replication through an evolutionary process. However, most of the identified genes encoding proteins with functional assignments were similar and homologous to genes found in other bacterial species, suggesting that both colony variants encode similar proteins essential for cellular maintenance and replication. Conversely, the number of genes that encode hypothetical proteins was markedly different between the two morphotype variants. The average nucleotide identity of the orthologous fragment pairs between the genomes of the large and small colony phenotypes was between 77.65 and 78.77%, and this warrants the classification of the small and large phenotypes into two different species [17]. In addition, a similar number and type of putative antimicrobial resistance genes have been identified between the three isolates; however, none were recognized as clinically relevant.

The only virulence-associated gene identified in the two large colony isolates of *A. hippocoleae* was the *UvrA* gene, which encodes for the excinuclease ABC subunit A protein that is part of the nucleotide excision repair (NER) pathway [34,35]. The NER pathway is the most important DNA repair system that enables the recognition and repair of any type of chemically damaged DNA base in bacteria [36]. UrvA protein contributes to the pathogenesis of bacterial pathogens by aiding bacteria to resist and adapt to acidic pH conditions and by promoting intracellular bacterial survival and replication. The UvrA protein has been identified and functionally characterized in different species of pathogenic bacteria, including *Helicobacter pylori* [37,38], *Streptococcus mutans* [39], *Arcanobacterium hemolyticum* [40], *Listeria monocytogenes* [41], *Mycobacterium tuberculosis* [42], and *Borrelia burgdorferi* [43]. Similarly, the UvrA protein could provide the survival of *A. hippocoleae* in the upper reproductive tract of mares where it can become acidic, especially during diestrus [44]. The protein may also protect the bacteria from phagocytic destruction because of exposure to reactive oxygen and nitrogen species. The *UvrA* gene was not detected in the small colony phenotype of *A. hippocoleae* by the PATRIC and Victor virulence gene databases. Interestingly, the inactivation of the NER protein UvrD1 in *Mycobacterium tuberculosis* resulted in a small colony phenotype. In addition, the UvrA-UvrD1 mutant of *M. tuberculosis* was markedly attenuated [34]. The *leuD* gene, which expresses a 3-isopropoylmalate dehydratase small subunit [35], was the virulence gene detected in the genome of the small colony variant in the PATRIC virulence gene database. Previously, a 3-isopropoylmalate dehydratase small subunit has been identified as part of the PhoPR system in *Mycobacterium avium* subsp. *paratuberculosis* (MAP) [35]. It has also been identified in the codY regulon in *Listeria monocytogenes* [45], which is an enzyme essential for leucine and complex lipid biosynthesis and contributes to oxidative stress response and virulence. The inactivation of the 3-isopropoylmalate dehydratase small subunit protein by gene mutation resulted in the inactivation of MAP [35,46] and *M. bovis* [47] with lower lesion severity in experimentally infected animals. It is interesting to identify a homolog of an important virulence determinant of intracellular bacteria in *A. hippocoleae*. However, both *UvrA* and *leuD* genes are present in many prokaryotic organisms as housekeeping genes and may have little relevance as virulence factors to *A. hippocoleae*. Therefore, functional studies are required to determine the significance of both proteins in morphotype switching and the virulence of *A. hippocoleae*. In addition, several hypothetical proteins were identified in the three isolates, and some might contribute to bacterial pathogenicity and require further study.

Moreover, a plasmid DNA was identified, which was carried by all three isolates of *A. hippocoleae*. The plasmid DNA had a sequence identity of 99.8% sequence identity with the pECQ4552_IHU08 plasmid, which was first reported in *Escherichia coli* strain Q4552 [48]. The plasmid does not encode notable virulence proteins or antimicrobial-resistant factors and does not contain mobility (*mob*) genes. However, the plasmid possesses genes that encode glycoside hydrolases, phage holin, and phage endopeptidase Rz and YlcI/YnfO family protein. Some of these proteins are involved in various cellular processes in virus-free bacteria, including programmed cell death [49,50], acetate metabolism [49], biofilm formation [51,52], DNA release [52], oxidative stress adaptation [51], and gene transfer [53,54]. The exact roles of these proteins in the cellular metabolism of *A. hippocoleae* are unknown. However, they may confer one or more of the above functions.

## 5. Conclusions

The 16S rRNA gene sequence-based identity between the large and small colony phenotypes of our bacterial isolates identified as *A. hippocoleae* by MALDI-TOF MS was greater than 99%. However, the whole genome-based sequence characterization revealed genetic differences between the two bacterial morphotypes, which warrants classification of the two isolates into two different species. Our study also provided baseline data, which may be used for determining the ability of the organism to cause disease in appropriate animal models and/or the role of the bacteria in the microbial ecology of the reproductive tract of mares.

## Figures and Tables

**Table 1:** Summary of annotated genome and protein features.

	Han-Large	Stan-Large	Stan-Small
Annotated Genome Features			
CDS	3234	2941	2399
Repeat regions	67	85	53
tRNA	46	46	45
rRNA	4	4	4
Partial CDS	0	0	0
Protein Features			
Hypothetical proteins	1799	1614	1126
Proteins with functional assignments	1435	1327	1273
Proteins with EC number assignments	652	597	590
Proteins with GO assignments	552	507	493
Proteins with pathway assignments	474	436	421
Proteins with PATRIC genus-specific family (PLfam) assignments	732	704	654
Proteins with PATRIC cross-genus family (PGfam) assignments	1151	1073	973

**Table 2:** Gene families used for phylogenetic construction are shown in Figure 4A. The gene families are ranked by alignment score (Align. Score) combining mean per-position variability (Mean Sqr Freq), alignment length (Align. Length), and gappiness (Prop Gaps). PGFam: PATRIC global protein families.

PGFam	Align. Score	Align. Length	Mean Sqr Freq	Prop Gaps	Product Used in the Analysis
PGF_02704551	22.49	1601	0.562	0.171	DNA-directed RNA polymerase beta’ subunit (EC 2.7.7.6) *rpoB*
PGF_10049811	18.27	1050	0.564	0.111	Protein translocase subunit *SecA*
PGF_05195027	16.30	496	0.732	0.033	ATP synthase beta chain (EC 3.6.3.14) *atpB*
PGF_09939762	13.87	593	0.570	0.149	SSU ribosomal protein *S1p*
PGF_10149521	12.57	439	0.600	0.052	Glucose-1-phosphate adenylyltransferase (EC 2.7.7.27) *glgC*
PGF_00421792	12.25	501	0.547	0.083	DNA repair protein *RadA*
PGF_00007028	12.10	551	0.515	0.135	Ribosome LSU-associated GTP-binding protein *HflX*
PGF_00007012	11.98	421	0.584	0.139	GTP-binding and nucleic acid-binding protein *YchF*
PGF_01937476	11.83	756	0.430	0.211	BioD-like N-terminal domain/Phosphate acetyltransferase (EC 2.3.1.8) *PTA*
PGF_03004613	10.20	488	0.462	0.107	Histidyl-tRNA synthetase (EC 6.1.1.21) *HisRS*
PGF_00634936	10.17	412	0.501	0.153	Protein QmcA (possibly involved in integral membrane quality control) *QmcA*
PGF_00912265	10.07	449	0.475	0.139	tRNA-dihydrouridine synthase *DusB*
PGF_04505269	10.01	322	0.558	0.165	SSU ribosomal protein S2p (*SAe*)
PGF_00016431	9.86	228	0.653	0.043	LSU ribosomal protein L3p (*L3e*)
PGF_02390924	9.86	367	0.515	0.099	16S rRNA (cytosine(1402)-N(4))-methyltransferase (EC 2.1.1.199) *rsmH*
PGF_04521913	9.75	561	0.412	0.146	UDP-N-acetylmuramoyl-tripeptide--D-alanyl-D-alanine ligase (EC 6.3.2.10) *murF*
PGF_00049896	9.48	237	0.616	0.088	SSU ribosomal protein S5p (*S2e*)
PGF_07063065	9.28	508	0.412	0.249	Transcription termination protein *NusA*
PGF_06162930	9.16	581	0.380	0.181	UDP-N-acetylmuramoyl-L-alanine--D-glutamate ligase (EC 6.3.2.9) *murD*
PGF_00419915	9.15	294	0.533	0.089	3-methyl-2-oxobutanoate hydroxymethyltransferase (EC 2.1.2.11) *panB*

## Data Availability

The sequence data have been deposited in the GenBank BioProject database with the following BioProject IDs: PRJNA1020882 (Stan-Large GenBank Accession# SAMN37533452)), PRJNA1020885 (Stan-Small GenBank Accession# SAMN37533502), and PRJNA1020887 (Han-Large GenBank Accession# SAMN37533508).

## Supplementary Materials

The following supporting information can be downloaded at https://www.mdpi.com/article/10.3390/ani14111609/s1, Table S1: Details of the source of samples for the isolation and identification of *A. hippocoleae*. Table S2: Genome assembly details, including genome statistics and quality. Table S3: A summary of specialty and AMR genes annotated in the genomes of the Han-Large, Stan-Large, and Stan-Small isolates of *A. hippocoleae*. Figure S1: Circa plot showing the percent identity between the protein sequences of our isolates and the protein sequences of seven different *Arcanobacterium* species. List of tracks, from outside to inside: *Arcanobacterium hippocoleae* DSM 15539, Stan-Small, Stan-Large, Han-Large, *Arcanobacterium* sp. JY-X174, *Arcanobacterium pinnipediorum* DSM 28752, *Arcanobacterium hemolyticum* strain NCTC9697, uncultured *Arcanobacterium* sp. strain SRR15732359, *Arcanobacterium pluranimalium* strain DSM 13483, and *Arcanobacterium canis* DSM 25104.
