# Supplementary material for: Genome Sequence Comparisons between Small and Large Colony Phenotypes of Equine Clinical Isolates of Arcanobacterium hippocoleae
Source: Animals (Basel). 2024 May 29;14(11):1609. doi: 10.3390/ani14111609 (PMC11171008; doi:10.3390/ani14111609)
Supplement: Supplementary file 1 [file animals-14-01609-s001.zip › animals-3009583-supplementary.pdf]

# Supplementary material

**Supplementary Table S1.** Details of the source of samples for the isolation and identification of *A. hippocoleae.*

| Animal ID | Breed | Symptoms | Sampling date | Province | Specimen site/type |
|---|---|---|---|---|---|
| **1** | Hanoverian | Placentitis and premature foaling the previous year. Purulent vaginal discharge and a small hyperchoic material in the uterus on ultrasound examination. | 17-03-2022 | Prince Edward Island (PEI), Canada | Vulval discharge/Swab |
| **2** | Thoroughbred | Treated with intrauterine penicillin. Successfully bred the previous year. | 26-04-2022 | Nova Scotia, Canada | Vagina/swab |
| **3** | Standardbred | None provided at the time of submission | 27-04-2022 | PEI | Uterus/swab |
| **4** | Standardbred | Pregnant, vaginal discharge and udder edema | 25-05-2022 | PEI | Uterus/lavage |
| **5** | Not specified | Pre-breeding swab. Mare did not conceive by AI the previous year. | 27-05-2022 | PEI | Uterus/swab |

**Supplementary Table S2.** Genome assembly details including genome statistics and quality.

| Genome Statistics | | | |
|---|---|---|---|
| | **Han-Large** | **Stan-Large** | **Stan-Small** |
| Contigs | 2 | 2 | 2 |
| Genome length contig 1 (circular) | 2420182 | 2434546 | 1988644 |
| Genome length contig 2 | 3566 | 3437 | 3359 |
| GC content | 48.29% | 48.36% | 48.46% |
| Contig L50 | 1 | 1 | 1 |
| Contig N50 | 2416616 | 2431109 | 1985285 |
| **Genome quality** | | | |
| Coarse Consistency | 94.9 | 95.1 | 94.8 |
| Fine Consistency | 88.7 | 90.9 | 89.4 |
| CheckM Completeness | 98.8 | 98.8 | 100 |
| CheckM Contamination | 22.4 | 14.7 | 14.3 |
| Genome Quality | Good | Good | Good |

**Supplementary Table S3.** A summary of specialty and AMR genes annotated in the genomes of Han-Large, Stan-Large and Stan-Small isolates of *A. hippocoleae*.

| **Specialty genes** | | | | |
|---|---|---|---|---|
| **Genes** | **Source** | **Han-Large** | **Stan-Large** | **Stan-Small** |
| Antibiotic resistance | CARD | 3 | 1 | 1 |
| Antibiotic resistance | PATRIC | 36 | 29 | 28 |
| Antibiotic resistance | NDARO | 2 | 0 | 0 |
| Drug target | DrugBank | 2 | 1 | 2 |
| Virulence factor | PATRIC_VF | 2 | 2 | 1 |
| Virulence factor | Victors | 1 | 1 | 0 |

| **Antimicrobial resistance (AMR) genes** | | | |
|---|---|---|---|
| **AMR Mechanism** | **Genes** | | |
| Antibiotic target in susceptible species | *alr, ddl, dxr, EF-G, EF-Tu, folA, dfr, folP, gyrA, gyrB, Iso-tRNA, murA, rho, rpoB, rpoC, s10p, s12p* | *alr, ddl, dxr, EF-G, EF-Tu, folA, dfr, folP, gyrA, gyrB, Iso-tRNA, murA, rho, rpoB, rpoC, S10p, s12p* | *alr, ddl, dxr, EF-G, EF-Tu, folA, dfr, folP, gyrA, gyrB, Iso-tRNA, murA, rho, rpoB, rpoC, s12p* |
| Antibiotic target protection protein | *lsa(C)* | - | - |
| Gene conferring resistance via absence | *gidB* | *gidB* | *gidB* |
| Protein altering cell wall charge conferring antibiotic resistance | *gdpD, pgsA* | *gdpD, pgsA* | *gdpD, mprF, pgsA* |
| Regulator modulating expression of antibiotic resistance genes | *mtrA, mtrB* | *mtrA, mtrB* | *mtrA, mtrB* |

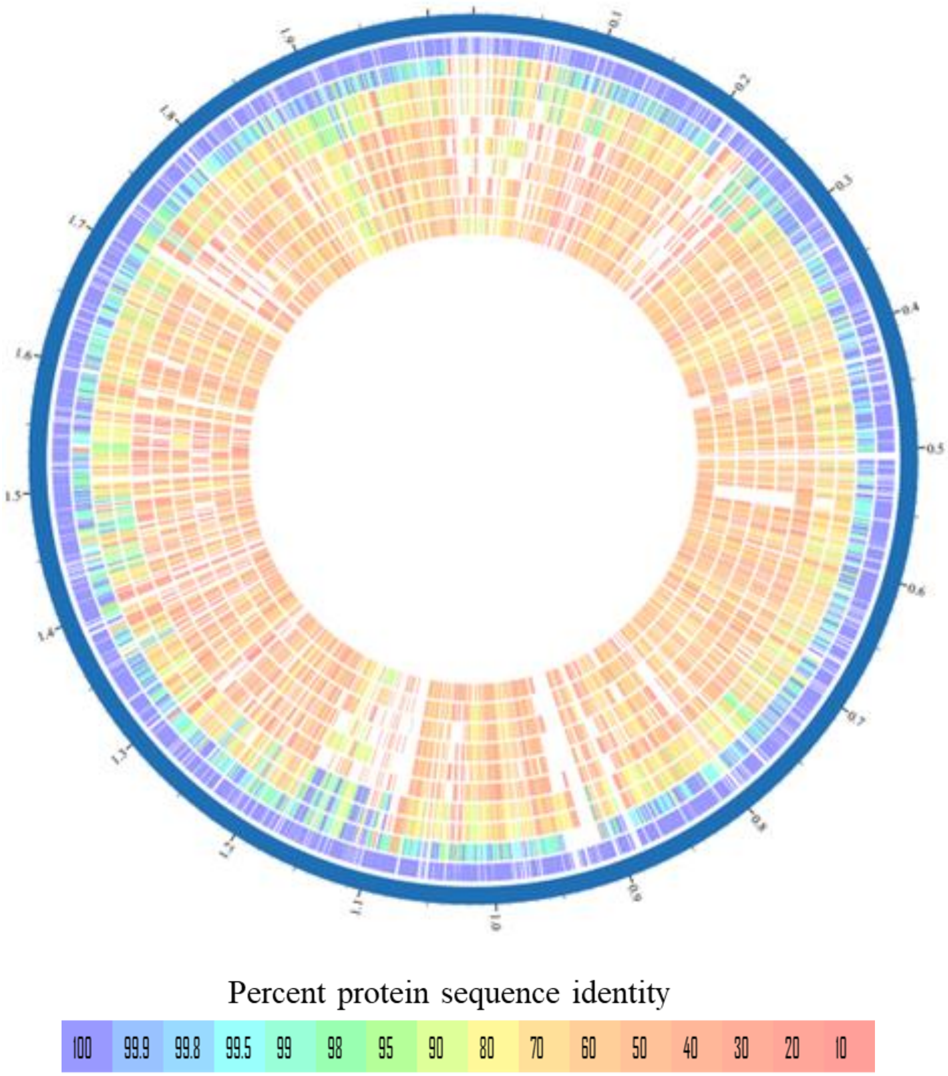


**Supplementary Figure S1**. Circa plot showing the percent identity between the protein sequences of our isolates and the protein sequences of seven different *Arcanobacterium* species. List of tracks, from outside to inside: *Arcanobacterium hippocoleae* DSM 15539, Stan-Small, Stan-Large, Han-Large, *Arcanobacterium* sp. JY-X174, *Arcanobacterium pinnipediorum* DSM 28752, *Arcanobacterium haemolyticum* strain NCTC9697, uncultured *Arcanobacterium sp*. strain SRR15732359, *Arcanobacterium pluranimalium* strain DSM 13483, *Arcanobacterium canis* DSM 25104.
